# Pericyte loss and endothelial activation define distinct vascular states in cerebral small vessel disease

**DOI:** 10.1007/s00401-026-03102-6

**Published:** 2026-10-13

**Authors:** A. Chagnot, D. Jaime Garcia, C. McQuaid, J. Cholewa-Waclaw, K. McDade, O. Dando, J. M. Wardlaw, C. Smith, A. Montagne

**Affiliations:** 1 Institute for Neuroscience and Cardiovascular Research (INCR), Chancellor’s Building, The University of Edinburgh, 49 Little France Crescent, Edinburgh, EH16 4SB UK; 2 Centre for Clinical Brain Sciences (CCBS), School of Clinical Sciences, College of Medicine and Veterinary Medicine, The University of Edinburgh, Edinburgh, EH16 4SB UK; 3 UK Dementia Research Institute (UK DRI) at the University of Edinburgh, Edinburgh, EH16 4SB UK; 4 British Heart Foundation - UK Dementia Research Institute (BHF-UK DRI) Centre for Vascular Dementia Research (CVDR) at the University of Edinburgh, Edinburgh, EH16 4SB UK; 5 Centre for Regenerative Medicine (CRM), Institute for Regeneration and Repair (IRR), The University of Edinburgh, Edinburgh, EH16 4UU UK; 6 Simons Initiative for the Developing Brain (SIDB), The University of Edinburgh, Edinburgh, EH8 9XF UK

**Keywords:** Cerebral small vessel disease, Pericytes, Capillaries, Ageing, Endothelial activation

## Abstract

Cerebral small vessel disease (cSVD) is a leading cause of stroke and vascular cognitive impairment, yet the cellular mechanisms underlying microvascular dysfunction in human disease remain incompletely understood. In particular, the relationship between pericyte alterations and endothelial activation, two key features of blood–brain barrier dysfunction, remains unresolved. Here, we performed a quantitative single-vessel analysis of the human cortical microvasculature across increasing cSVD severity and ageing. Using multiplex immunohistochemistry combined with spectral unmixing and automated image analysis, we analysed 11,409 microvascular fragments from post-mortem brain tissue derived from 20 cases. Endothelial cells, pericytes, and endothelial activation were assessed using PECAM-1, PDGFRβ, and VCAM-1, respectively. Microvascular density and diameter differed between cortical grey matter and the underlying white matter, with white matter vessels being less dense and wider in controls. While vessel diameter remained stable across cSVD severity groups, microvascular density increased with cSVD severity and age in the white matter. At the molecular level, PDGFRβ signal decreased markedly with increasing cSVD severity, consistent with reduced pericyte coverage. This reduction was observed in both grey and white matter and correlated with disease severity and age. Notably, intermediate severity groups displayed marked heterogeneity, with vessels exhibiting either preserved or markedly reduced PDGFRβ signal. In parallel, endothelial markers PECAM-1 and VCAM-1 increased significantly with disease severity, reflecting endothelial activation. Unsupervised Gaussian mixture clustering of marker expression identified three vascular states characterised by (i) preserved PDGFRβ signal with low endothelial activation, (ii) reduced PDGFRβ signal without endothelial activation, and (iii) reduced PDGFRβ signal combined with endothelial activation. These clusters broadly aligned with neuropathological severity but revealed heterogeneity not captured by post-mortem diagnosis alone. Together, these findings suggest that pericyte alterations and endothelial activation are partially dissociated processes in human cSVD and identify distinct microvascular phenotypes across disease severity. These cross-sectional observations provide a framework for future longitudinal and experimental studies to determine their temporal and mechanistic relationships.

**Supplementary Information:**

The online version contains supplementary material available at https://doi.org/10.1007/s00401-026-03102-6.

## Introduction

Cerebral small vessel disease (cSVD) is a leading cause of stroke and the principal vascular contributor to cognitive impairment and dementia. It affects the brain’s perforating arterioles, capillaries, and venules, and is characterised by a spectrum of radiological and pathological features including white matter hyperintensities (WMH), lacunes, microbleeds, and blood–brain barrier (BBB) dysfunction [51]. Despite its high prevalence and clinical impact, there are currently no disease-modifying therapies, although recent clinical trials targeting cSVD mechanisms have shown promise, highlighting an incomplete understanding of the cellular mechanisms that initiate and propagate microvascular injury [32, 52].

At the capillary level, the cerebral microvasculature forms a key part of the neurogliovascular unit (NGVU), a multicellular system in which endothelial cells and pericytes maintain BBB integrity and regulate vascular function. Endothelial cells form the structural basis of the BBB and regulate molecular transport, immune surveillance, and vascular tone. Pericytes, embedded within the capillary basement membrane, provide structural support, regulate capillary diameter to control blood flow, and support endothelial stability through platelet-derived growth factor-B/platelet-derived growth factor receptor-β (PDGF-BB/PDGFRβ) signalling [2, 11]. Disruption of endothelial-pericyte crosstalk is increasingly recognised as a central feature of microvascular dysfunction in ageing and neurodegenerative disease [1, 8, 41].

Endothelial activation is a consistent hallmark of cSVD and represents a shift towards a pro-inflammatory and pro-thrombotic phenotype. Upregulation of adhesion molecules such as vascular cell adhesion molecule-1 (VCAM-1), intercellular adhesion molecule-1 (ICAM-1), and selectins promotes leukocyte adhesion, vascular inflammation, and BBB disruption [21, 47, 51]. Neuroimaging studies further demonstrate associations between endothelial activation and impaired cerebrovascular reactivity, reduced cerebral blood flow (CBF), and increased BBB permeability [6, 23, 48]. Circulating biomarkers of endothelial activation have also been linked to cSVD burden and progression, although findings remain heterogeneous and mechanistic interpretation is limited by the lack of spatial resolution [22]. For example, soluble VCAM-1 has been identified as a circulating mediator of ageing-associated cognitive decline, linking vascular inflammation to brain dysfunction [53]. More recently, large-scale proteomic studies have revealed both shared and distinct molecular signatures across different radiological manifestations of cSVD, highlighting the biological heterogeneity of the disease and the involvement of multiple vascular wall compartments [19]. In parallel, biofluid studies provide further evidence of pericyte injury in ageing and Alzheimer’s disease (AD), with increased levels of soluble PDGFRβ in cerebrospinal fluid associated with BBB dysfunction, vascular damage, and cognitive decline [33, 35, 36].

Pericyte dysfunction has also emerged as a key contributor to microvascular pathology. Experimental models consistently demonstrate that pericyte loss leads to reduced CBF, endothelial destabilisation, and BBB breakdown [4, 16]. Mechanistically, disruption of pericyte-endothelial signalling induces a pro-inflammatory endothelial phenotype, including upregulation of VCAM-1 and ICAM-1, increased leukocyte adhesion, and altered vascular homeostasis [30, 37, 49]. These findings support a model in which pericytes actively restrain endothelial activation under physiological conditions.

However, whether pericyte loss is a consistent and defining feature of human ageing and cSVD remains unresolved. Several neuropathological studies report reduced pericyte coverage and microvascular alterations in AD and other dementias, particularly in white matter regions [13, 18, 43]. In contrast, other studies report preserved pericyte coverage, or even evidence of microvascular remodelling without overt pericyte loss in AD [15, 34]. Recent transcriptomic analyses further highlight marked heterogeneity in vascular cell states across sporadic and familial AD, suggesting that pericyte alterations are context-dependent and may vary with disease burden, vascular compartment, and pathological subtype rather than representing a uniform loss [44].

These discrepancies highlight a central unresolved question: whether pericyte alterations represent a primary driver of microvascular dysfunction, a secondary consequence of endothelial injury, or part of a dynamic and heterogeneous vascular response in disease. Importantly, many existing studies rely on bulk or region-averaged analyses, which may obscure heterogeneity at the level of individual microvessels and contribute to conflicting findings.

Consistent with this, cSVD is increasingly recognised as a disorder of microvascular function and cellular composition rather than a disease primarily defined by remodelling of larger vascular branches such as arteries and veins. Structural features such as vessel density and diameter are often relatively preserved, including in cases with lower disease burden, suggesting that cellular and molecular alterations within intact vascular networks are key features of pathology [40, 41]. Recent advances in multiplex imaging and quantitative pathology now enable detailed interrogation of the cerebral microvasculature at single-vessel resolution. These approaches allow simultaneous assessment of endothelial and perivascular markers, providing an opportunity to resolve the relationship between pericyte alterations and endothelial activation across cSVD severity.

In this study, we investigate how pericyte and endothelial activation markers relate in cSVD. By applying high-resolution quantitative analyses to human brain tissue spanning a range of neuropathological cSVD severity, we aim to define the extent and distribution of pericyte marker alterations, characterise endothelial marker expression, and determine how these features co-vary within the brain microvasculature.

## Methods

### Human tissue acquisition, study cases, and clinical assessment

Brain sections in Brodmann area 4 including the premotor cortex and the underlying white matter were obtained from 20 cases. The tissue originated from three large Scottish cohorts: the Lothian Birth Cohort (LBC) [12], the Lothian study of Intracerebral Haemorrhage, Pathology, Imaging and Neurological outcome (LINCHPIN) [42], and the Edinburgh Sudden Death Brain Bank [46]. The Scotland A Research Ethics Committee approved this study (10/MRE00/23). The Edinburgh Sudden Death Brain Bank is funded by the UK Dementia Research Institute and was established in 2003 to increase access to younger control cases. All sudden deaths require a post-mortem examination by the Procurator Fiscal and Forensic Pathology Department who identify suitable cases of presumed healthy brains; most sudden death cases are sudden cardiac deaths. The Edinburgh Brain Bank functions within the Human Tissue (Scotland) Act of 2006 and has full ethical approval for the use of brain tissue in research (East of Scotland Research Ethics Service, ref 16/ES/0084). Informed consent was obtained from all participants in the LBC and LINCHPIN studies, and post-mortem authorisation was obtained from next of kin in all cases.

Relevant clinical information, demographics, and clinical history were collected by a trained research nurse. In sudden death cases this was extracted retrospectively from GP or hospital records. In LBC36 and LINCHPIN, the data were collected prospectively as part of the original LBC and LINCHPIN studies. Data included participant age at death, sex, prior diagnoses of hypertension, diabetes, hypercholesterolemia, heart disease, or atrial fibrillation, confirmed cause of death, post-mortem interval, and any relevant past medical history, including chronic disease, malignancy, stroke, inflammatory or rheumatologic disease, psychiatric illness, or cardiovascular disease (Table 1). Exclusion criteria included a post-mortem interval greater than five days and a known diagnosis of neurological or neurodegenerative disease other than cSVD.

**Table 1:**
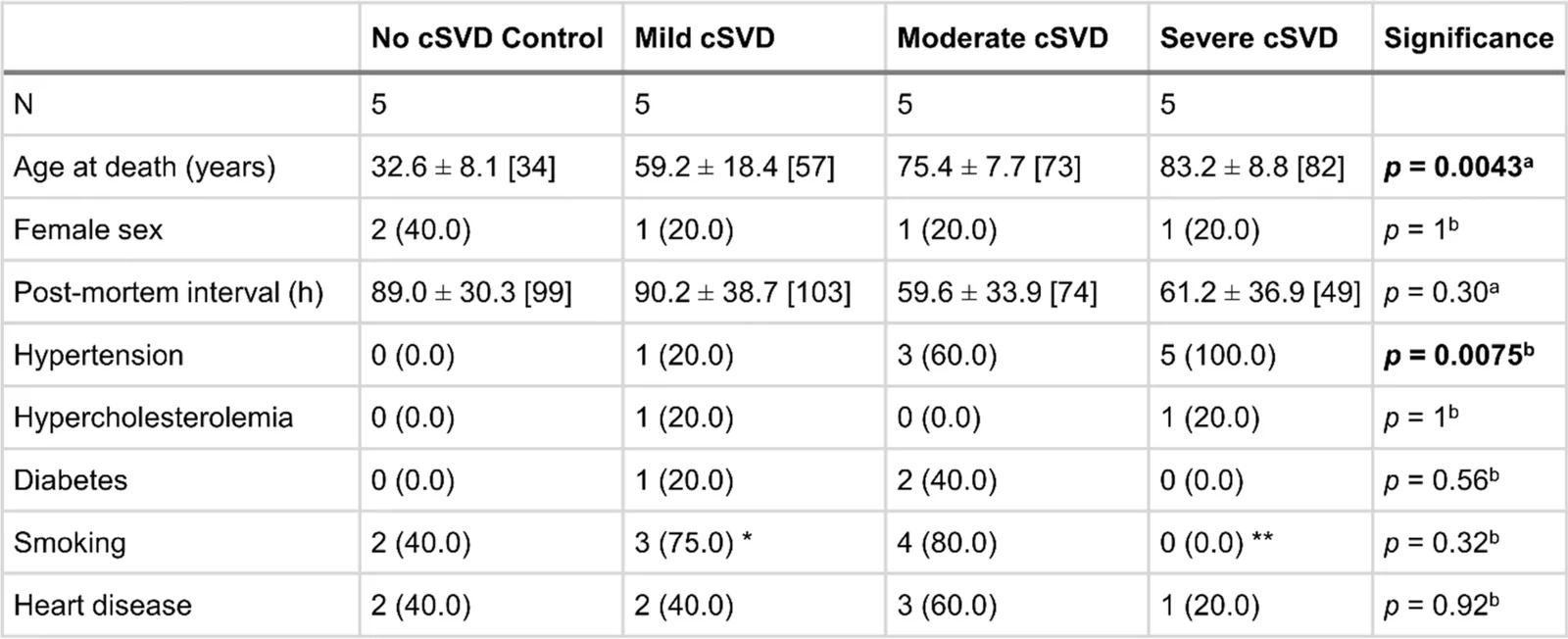
Sample characteristics

Brain tissue from 20 donors was included in this study. Age and post-mortem interval are reported as mean ± standard deviation (SD) [median]. Other values are reported as n (%). cSVD, cerebral small vessel disease. Group-wise significance was assessed using Kruskal–Wallis (a) or Fisher’s exact (b) tests. *, one missing value; **, three missing values

### Tissue preparation

Brain tissue processing is described in the respective LBC [12], LINCHPIN [42], and Edinburgh Sudden Death Brain Bank [46] protocols. Paraffin-embedded blocks of brain tissue were fixed using unbuffered 10% formalin for 48–72 h prior to histological processing. Formalin-fixed, paraffin-embedded Sects. (4 μm thick, ~ 2 cm^2^) in the primary motor cortex / precentral gyrus (Brodmann area 4) were selected to include both grey and white matter.

### cSVD assessment

There are no agreed, validated protocols for cSVD assessment. Neuropathological examination [20, 45] was carried out by an experienced neuropathologist (Prof. Colin Smith), blinded to clinical, radiographic, and genetic data. Cerebral small vessel disease (cSVD) burden, graded as no cSVD control (n = 5), mild cSVD (n = 5), moderate cSVD (n = 5), or severe cSVD (n = 5), was based on the presence and severity of vessel wall sclerosis (collagen deposition with loss of smooth muscle cells), increased vessel wall thickness, reduced lumen diameter, enlarged perivascular spaces, perivascular haemosiderin deposition, white matter rarefaction, lacunar infarcts, and microhaemorrhages [20, 45]. Two cases in the severe cSVD group also showed mild cerebral amyloid angiopathy on neuropathological examination.

### Immunohistochemistry

Experimenters were blinded to cSVD severity score, cohort of origin and all in vivo data. Immunohistochemistry was performed using the BOND RX Fully Automated Research Stainer (Leica Biosystems). Tissue sections mounted on slides were covered with BOND Universal Covertile (Leica Biosystems S21.4611) and processed using a Polymer Detection Kit (Leica Biosystems DS9800) and an HRP PLEX Kit (Leica Biosystems DS9914), which includes a base platform comprising peroxide block, post-primary reagent, polymer reagent, DAB chromogen, and haematoxylin counterstain, as well as the required chromogens green (Leica Biosystems DC9913) and red (Leica Biosystems DS9390).

Primary antibodies were applied following antigen retrieval using BOND Epitope Retrieval Solution 1 (Leica Biosystems AR9961). For endothelial cells, PECAM-1 (Abcam ab134168, 1:250 dilution; 20 min retrieval); for the pericytes, PDGFRβ (Abcam ab69506, 1:250 dilution; 20 min retrieval); for endothelial activation, VCAM-1 (Abcam, ab134047, 1:500 dilution; 30 min retrieval); and for microglia, Iba1 (Abcam ab178846, 1:3000 dilution; 30 min retrieval) were used. One set of sections received a combination of PDGFRβ + PECAM-1 + VCAM-1, while a second set received a combination of Iba1 + PECAM-1 + VCAM-1. A total of 150 μL of antibody solution in Leica antibody diluent (Leica Biosystems AR9352) was applied per slide, after which the instrument executed the sequential multiplex staining protocol.

### Multispectral imaging and microscopy

Aged human post-mortem tissue can exhibit substantial autofluorescence, including lipofuscin, which can complicate conventional immunofluorescence. We therefore used chromogenic multiplex staining combined with multispectral imaging. Brightfield images were acquired across 20 wavelengths and spectrally unmixed into marker-specific pseudo-channels using reference spectra generated from single-stained tissue from the same cohort. This enabled separation of chromogens with partially overlapping visual appearances and provided individual channels for quantitative image analysis (Supplementary Fig. 1) [29].

Stained tissue sections were imaged at 20 × magnification using the PhenoImager HT Automated Quantitative Pathology Imaging System (Akoya Biosciences). For each individual case, eight to ten fields of view (929.74 × 697.31 μm area, 0.5 × 0.5 μm in-plane resolution), equally distributed between white and grey matter, were selected for multispectral imaging and spectral unmixing. Briefly, light from brightfield images was unmixed using a liquid–crystal tuneable band-pass filter (420, 440, 460, 480, 500, 520, 540, 560, 580, 600, 620, 640, 660, 680, 700, 720, 740, 760, 780 and 800 nm) and compared with the spectral signatures of the individual chromogens (Vector Red, Methyl Green, Haematoxylin and DAB). An in-house spectral unmixing algorithm was then applied to isolate individual stain signals, yielding separate pseudo-fluorescent channels, enabling further processing of the image analogous to fluorescence microscopy [29].

### Vascular segmentation

**Fig. 1:**
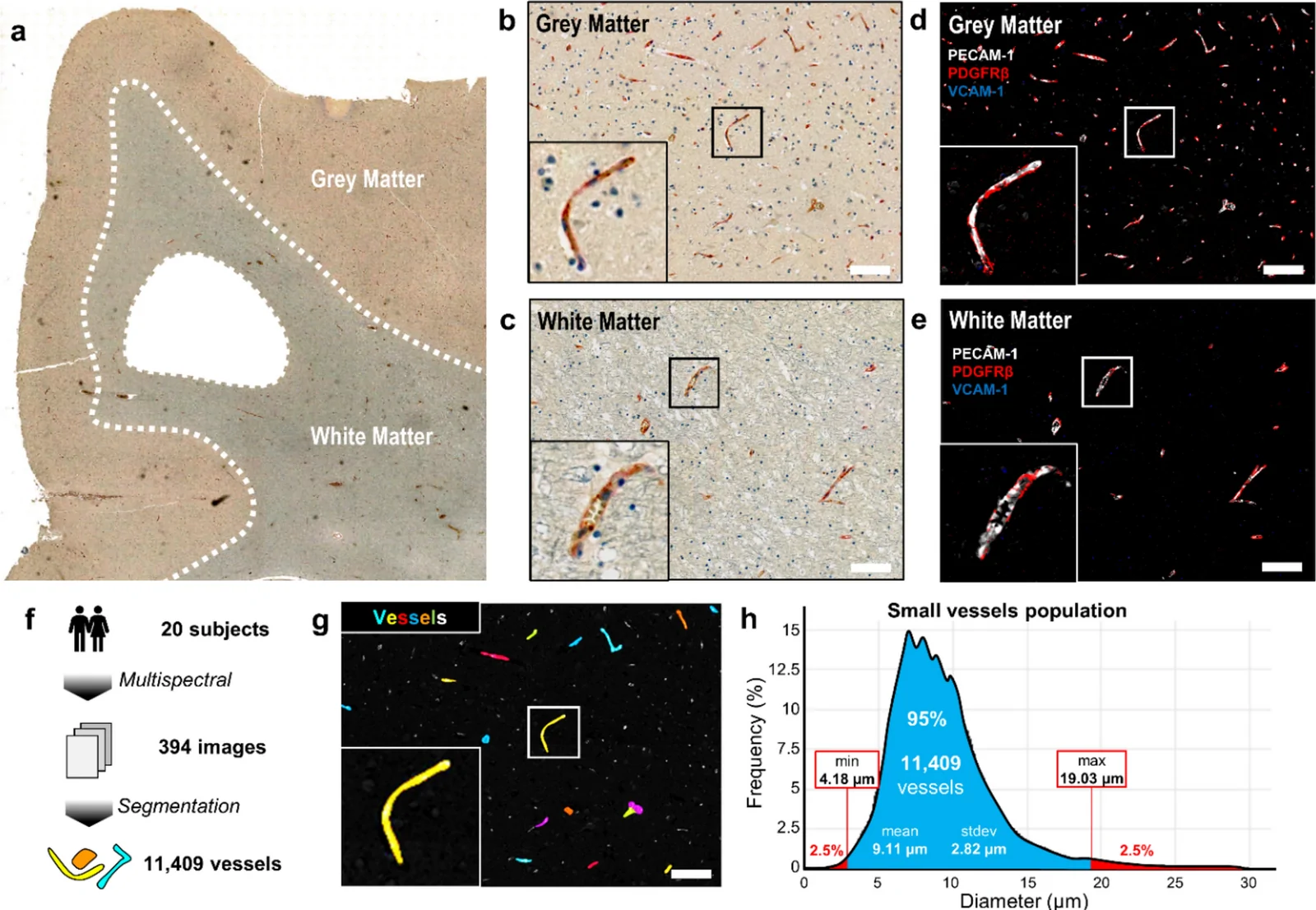
Extraction of microvascular features from multiplex brightfield imaging. **a** Representative brightfield image of a cortical brain section (Brodmann area 4, primary motor cortex) from a 34-year-old male no cSVD control, with annotated grey and white matter sampling regions. A minimum of eight fields of view (930 × 700 μm) were analysed per case. **b**, **c** Representative brightfield fields of view and corresponding vessel close-ups in grey (**b**) and white (**c**) matter. **d**, **e** Pseudo-fluorescent reconstructions of the images in (**b**) and (**c**) showing separated chromogenic signals for PECAM-1 (DAB), PDGFRβ (VectorRed), and VCAM-1 (MethylGreen). **f** Overview of the image processing and vascular segmentation workflow implemented in the MATLAB-based Morph-O-Matic[9] pipeline. **g** Segmentation of individual vascular fragments based on the PECAM-1 signal. **h** Diameter distribution of segmented vascular fragments; extreme values were excluded to remove segmentation artefacts. Scale bars: 200 μm. DAB, 3,3’-diaminobenzidine; PECAM-1, platelet endothelial cell adhesion molecule-1; PDGFRβ, platelet-derived growth factor receptor-β; VCAM-1, vascular cell adhesion molecule-1

Microvascular features were extracted using a brightfield-based multiplex immunohistochemistry and quantitative image analysis pipeline (Fig. 1). Grey and white matter regions were defined in the primary motor cortex (Brodmann area 4) (Fig. 1a). Representative fields of view and individual vessel close-ups illustrate the distinct microvascular architecture in grey and white matter (Fig. 1b, c). Chromogenic signals corresponding to PECAM-1 (DAB), PDGFRβ (VectorRed), and VCAM-1 (MethylGreen) were separated into distinct pseudo-fluorescent channels by spectral unmixing (Fig. 1d, e). Blood vessels were segmented based on the PECAM-1 signal using Morph-O-Matic [9], a MATLAB-based (MATLAB R2021b, The MathWorks Inc.) tool (Fig. 1f, g). The PECAM-1 signal was processed using the following steps: thresholding above the 9th decile of image intensity, removal of particles smaller than 125 μm^2^, 1.5 μm dilation, 7.5 μm median smoothing, filling of holes smaller than 750 μm^2^, 1 μm erosion, and a final 2.5 μm median smoothing. Eight-connected logic was used to separate the resulting vascular objects, which were subsequently analysed independently. To reduce bias in signal quantification, PDGFRβ and VCAM-1 coverage, thresholded above their respective mean signal intensities, was calculated within PECAM-1-positive vascular objects. Metrics extracted from each vascular object included length, vessel-wide diameter, area, PECAM-1 signal intensity, PDGFRβ signal intensity, and VCAM-1 signal intensity.

The dataset was refined by excluding extreme vessel diameter values to remove segmentation artefacts smaller than the 2.5th quantile (4.18 μm) and larger than the 97.5th quantile (19.03 μm) leaving a total pool of vessels of 11,409 individual objects (mean diameter 9.11 μm, standard deviation 2.82 μm) from 20 cases (Fig. 1h). For each of the 394 (8 to 10 per case and per matter, white or grey) fields of view, vascular density, averaged vascular diameters, average PECAM-1 signal intensity, average PDGFRβ signal intensity, and average VCAM-1 signal intensity were computed. All the averages were weighted by the length of individual vascular objects.

### Microglial segmentation

As for the vascular segmentation, microglial features were segmented using Morph-O-Matic. The Iba1 signal was thresholded using Otsu’s method [39], then the total covered area, number of particles and median size of particles were processed. On 4 μm sections such as the ones used in this article, it is not possible to observe entire microglia cells. Consequently, we used the number of segmented particles and their median area as proxy descriptors for microglial ramification. As microglia extend in 3 dimensions, the 4-μm slice only captures a fraction of the cell, hence the fragmented appearance of highly ramified microglia (Supplementary Fig. 2b) [54].

### Statistical analyses

Processing of the data was done using R version 4.2.0 (The R Core Team, 2022). Vessels with extreme diameters were excluded to minimise the inclusion of segmentation artefacts. Kruskal–Wallis or Fisher’s exact tests were computed on the epidemiological data presented in Table 1 to investigate inter-group differences. Mean, standard deviation, and median of each metric were reported for each disease group and matter in Table 2. Log transform was applied to the vascular density and signal data to adjust to normal distribution. Twenty-seven outliers (of 394 images) were identified using Mahalanobis distance (p = 0.05) across all five metrics and excluded.

**Table 2:**
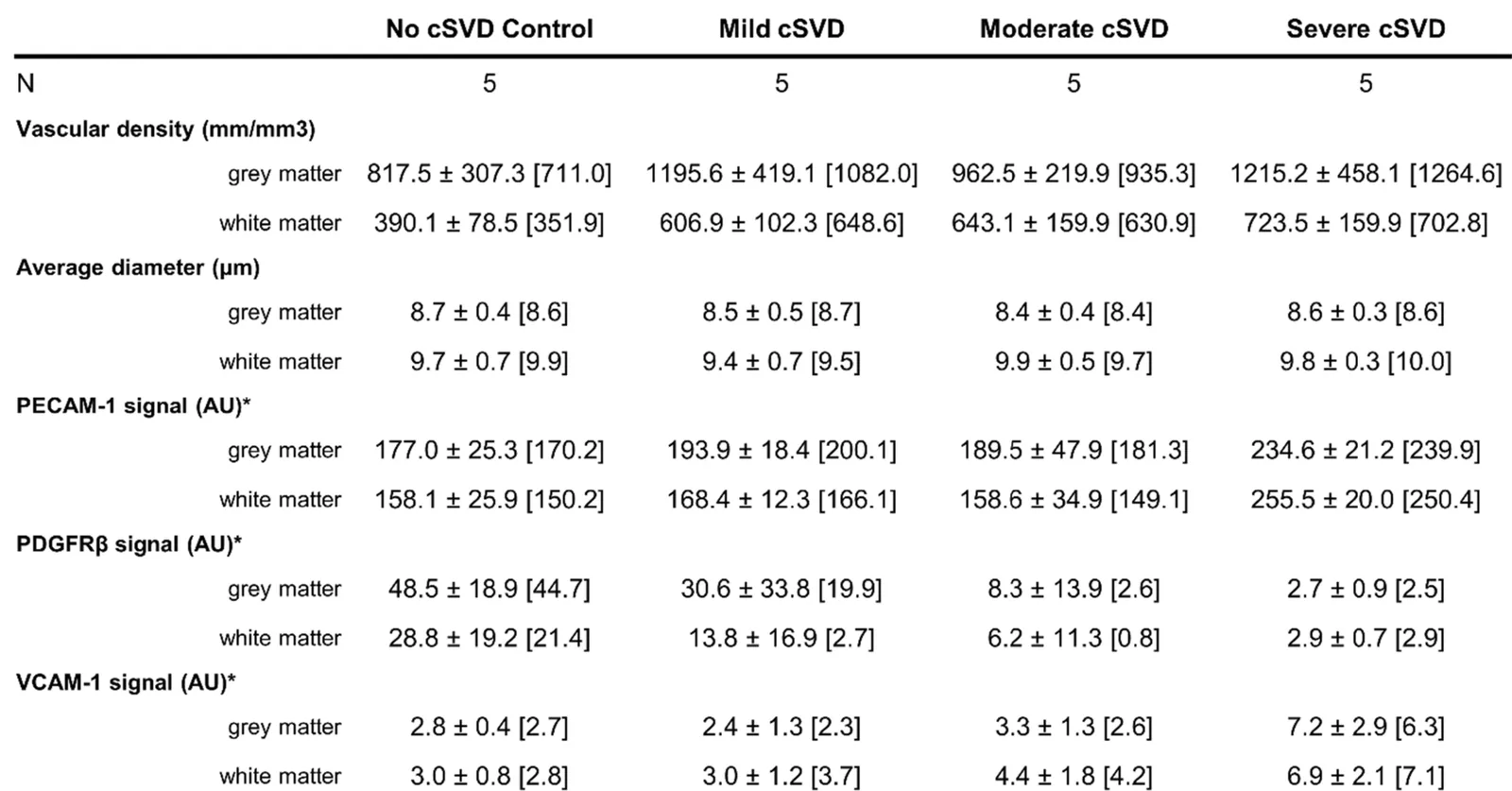
Quantitative vascular metrics by diagnostic group

All metrics are reported as mean ± standard deviation (SD) [median]. AU, arbitrary units; cSVD, cerebral small vessel disease; PECAM-1, platelet endothelial cell adhesion molecule-1; PDGFRβ, platelet-derived growth factor receptor-β; VCAM-1, vascular cell adhesion molecule-1. *, values multiplied by 10^3^ for display

Linear mixed-effects models assessed per-field of view averaged vascular diameter and log-transformed per-field of view averaged vascular density, PECAM-1, PDGFRβ, and VCAM-1 signal using the lme4 R package[3], with cSVD group and tissue compartment (grey vs white matter) included as fixed-effect covariates. A case random effect was included to account for multiple non-independent measurements within each case, and models were formulated as follows:

variable∼diagnosis∗matter+(1|case)

Here, variable was replaced with vascular diameter, log vascular density, log PECAM-1 signal, log PDGFRβ signal, and log VCAM-1 signal. ANOVA followed by estimated marginal means assessed the significance of group differences using the car and emmeans R package [27].

Gaussian mixtures modelling approach was used to identify potential patterns across the three markers PECAM-1, PDGFRβ, and VCAM-1. Log-transformed measures were scaled then processed with the mclust R package. Silhouette plots and PERMANOVAs were used to evaluate how well the diagnosis and markers-based clustering separated the cases. Statistics on the markers-based clusters were performed similarly to those based on the diagnostic groups with linear mixed-effect models and are presented in Supplementary Fig. 1 and Table 3.

**Table 3:**
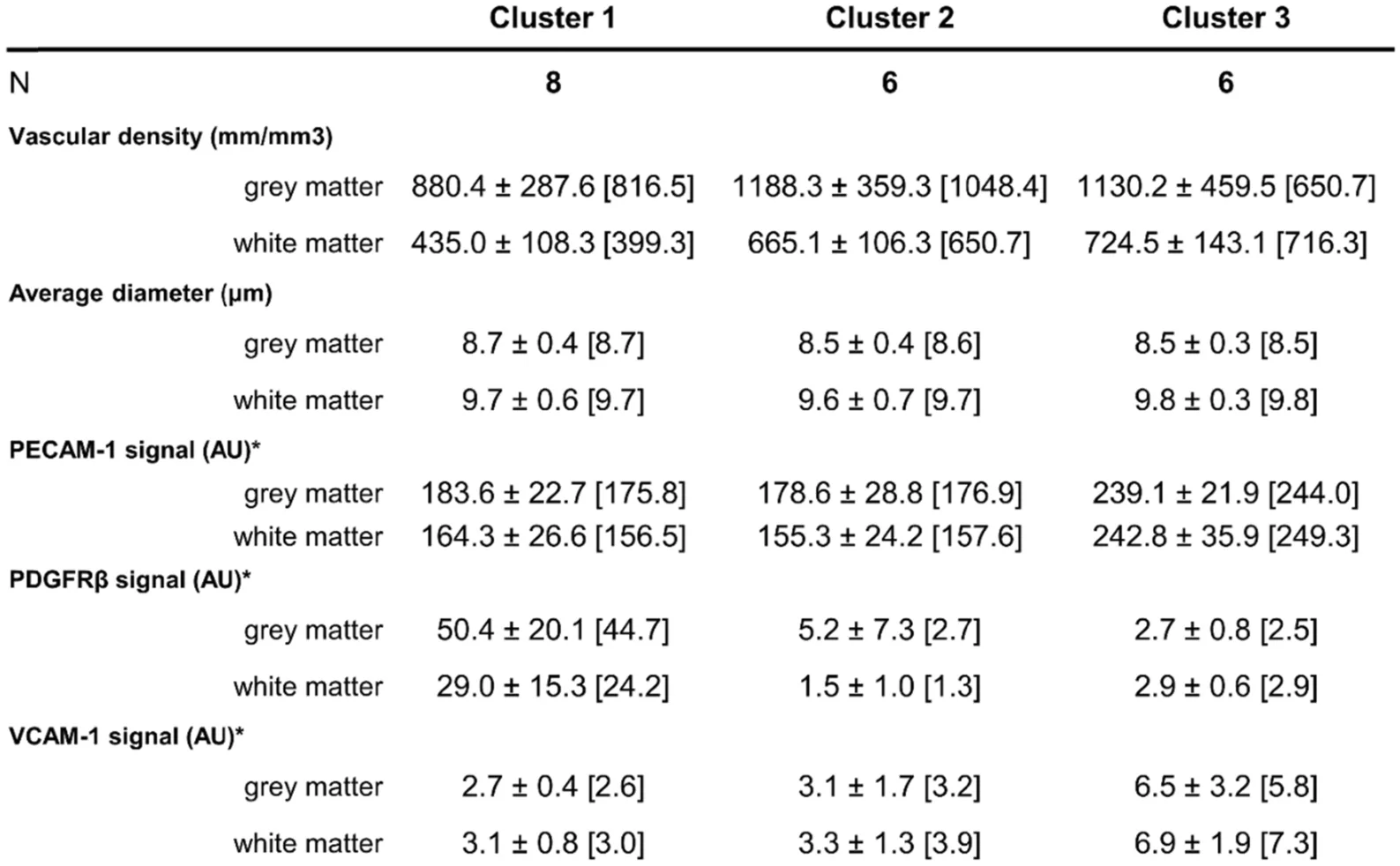
Quantitative vascular metrics by marker-based cluster

All metrics are reported as mean ± standard deviation (SD) [median]. AU, arbitrary units; PECAM-1, platelet endothelial cell adhesion molecule-1; PDGFRβ, platelet-derived growth factor receptor-β; VCAM-1, vascular cell adhesion molecule-1. *, values multiplied by 10^3^ for display

### Blood biomarkers analysis

ELISA readouts for blood VCAM-1 and PDGF-BB, Fazekas scores, and status for hypercholesterolemia, diabetes, and hypertension were obtained from a previously published cohort of lacunar stroke patients recruited from the Lothian stroke services between August 2018 and December 2021 (Supplementary Table 1) [23].

Half-maximal PDGF-BB and VCAM-1 values were used to define four exploratory quadrants: low VCAM-1/high PDGF-BB (V-/P + , n = 41), low VCAM-1/low PDGF-BB (V-/P-, n = 99), high VCAM-1/low PDGF-BB (V + /P-, n = 34), and high VCAM-1/high PDGF-BB (V + /P + , n = 5) (Supplementary Fig. 4a). The V + /P + group was not included in subsequent group comparisons because of its small size. Age comparisons were adjusted for hypertension, diabetes, and hypercholesterolaemia, while Fazekas score comparisons were additionally adjusted for age.

## Results

### Demographic comparisons

There were no differences in sex (5/15 females/males, p = 1.00), post-mortem interval (p = 0.30), hypercholesterolemia (p = 1.00), diabetes (p = 0.56), smoking status (p = 0.32), or heart disease (p = 0.92) between no SVD controls and increasing cSVD severity groups (Table 1). However, age at death increased with cSVD severity (no SVD controls: 32.6 ± 8.1 years; mild cSVD: 59.2 ± 18.4 years; moderate cSVD: 75.4 ± 7.7 years; severe cSVD: 83.2 ± 8.8 years; p = 0.0043), consistent with the known age association of cSVD. Hypertension, one of the strongest risk factors for cSVD, was also found to increase in prevalence across disease groups (p = 0.0075).

### Microvascular density, but not diameter, correlates with cSVD severity and age at death

**Fig. 2:**
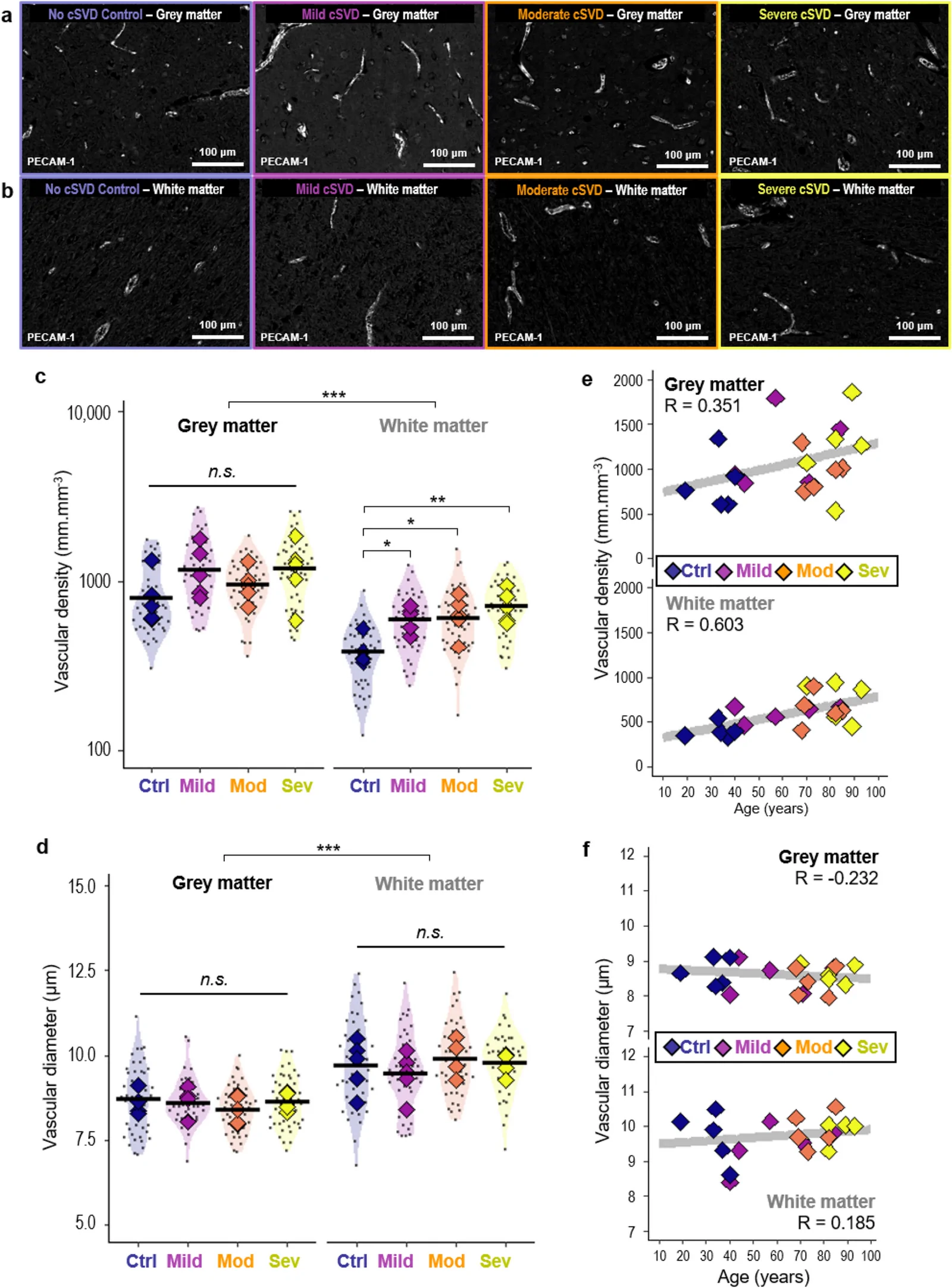
Cortical microvascular morphology across cSVD severity. **a**, **b** Representative PECAM-1 pseudo-fluorescent images in grey (**a**) and white (**b**) matter across cSVD severity groups (control, mild, moderate, severe). **c** Vascular density (vascular length per tissue volume) by group in grey and white matter. **d** Vascular density plotted against age at death. **e** Mean microvascular diameter by group in grey and white matter. **f** Microvascular diameter plotted against age at death. Diamond-shaped data points represent individual cases, whereas smaller dots represent analysed images. Scale bars: 100 μm. Linear mixed-effects modelling was used to account for case-level variability, with tissue compartment (grey/white matter) and images included as hierarchical factors. ANOVA on the fitted model identified significant differences between grey and white matter for vascular density and vascular diameter. Post-hoc comparisons were performed using estimated marginal means (emmeans), revealing significant differences in vascular density between cSVD groups in white matter (n.s., not significant; *, p-value < 0.05; **, p-value < 0.01; ***, p-value < 0.001). Pearson’s correlation coefficients (R) are shown in panels D and F. cSVD, cerebral small vessel disease; Ctrl, no cSVD control; Mild, mild cSVD; Mod, moderate cSVD; Sev, severe cSVD; PECAM-1, platelet endothelial cell adhesion molecule-1

Analyses were restricted to microvessels (mean diameter: 9.1 ± 2.8 μm), representing the most abundant vascular bed in brain tissue. Marked morphological differences were observed between tissue compartments, consistent with previous observations. In the no SVD control group, white matter vessels were 51% less dense (Fig. 2a, b, c) and 14% wider (Fig. 2a, b, e) than grey matter vessels.

While vascular density showed a trend towards increasing with cSVD status in both tissue compartments, it was statistically significant only in white matter, where the density of vessels doubled in severe cSVD cases compared to no cSVD controls (Fig. 2c, p= 0.0017) and increased with age (Fig. 2d, Pearson’s R = 0.603). Conversely, microvascular diameter did not change significantly across cSVD severity groups (Fig. 2e) or with age (Fig. 2f).

### Microglial morphology is altered in severe cSVD

On visual inspection, microglia appeared more amoeboid in the severe cSVD group compared with the no cSVD, mild and moderate groups (Supplementary Fig. 2a). Iba1-positive area and the number of segmented particles were respectively 24% and 46% lower in grey matter from severe compared with mild cSVD cases (p = 0.022 and p = 0.010; Supplementary Fig. 2c, d), with no significant differences detected between the other groups. Median particle area was increased in severe cSVD compared with no cSVD and mild cSVD in grey matter (60% and 54%, respectively; p = 0.015 and p = 0.030), and compared with no cSVD in white matter (54%; p = 0.041; Supplementary Fig. 2e).

### VCAM-1 and PECAM-1 expression are markedly increased in the cortex of cases with severe cSVD

**Fig. 3:**
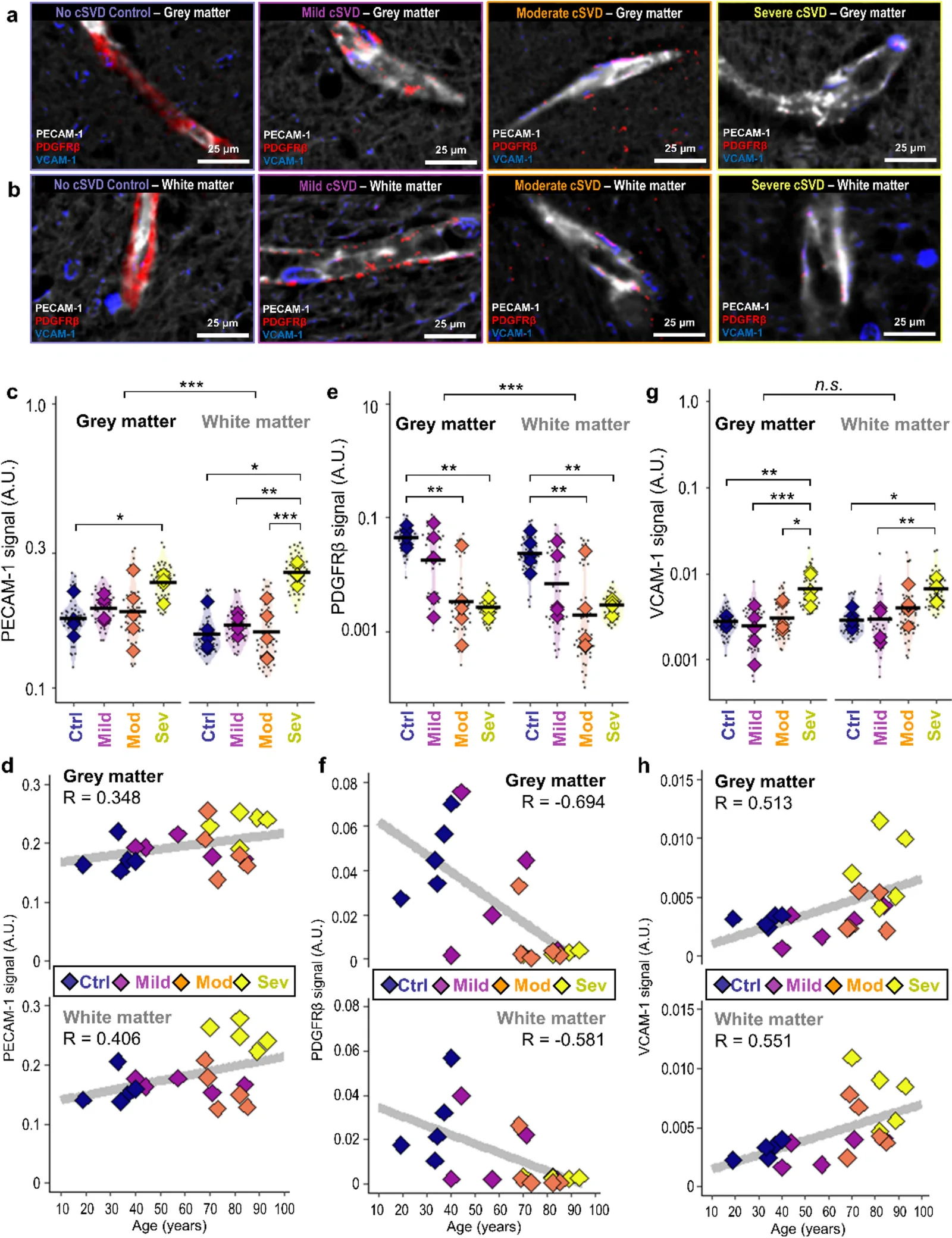
PECAM-1, PDGFRβ, and VCAM-1 signals across cSVD severity. **a**,** b** Representative microvessel close-ups in grey (**a**) and white (**b**) matter showing PECAM-1 (grey), PDGFRβ (red), and VCAM-1 (blue) across cSVD severity groups (control, mild, moderate, severe). **c** PECAM-1 signal by group in grey and white matter. **d** PECAM-1 signal plotted against age at death. **e** PDGFRβ signal by group in grey and white matter. **f** PDGFRβ signal plotted against age at death. **g** VCAM-1 signal by group in grey and white matter. **h** VCAM-1 signal plotted against age at death. Diamond-shaped data points represent individual cases, whereas smaller dots represent analysed images. Scale bars: 25 μm. Linear mixed-effects modelling was used to account for case-level variability, with tissue compartment (grey/white matter) and images included as hierarchical factors. ANOVA on the fitted model identified significant differences between grey and white matter for PECAM-1 and PDGFRβ signals. Post-hoc comparisons were performed using estimated marginal means (emmeans) to test differences between cSVD severity groups (n.s., not significant; *, p-value < 0.05; **, p-value < 0.01; ***, p-value < 0.001). Pearson’s correlation coefficients (R) are shown in panels D, F, and H. AU, arbitrary units; cSVD, cerebral small vessel disease; Ctrl, no cSVD control; Mild, mild cSVD; Mod, moderate cSVD; Sev, severe cSVD; PECAM-1, platelet endothelial cell adhesion molecule-1; PDGFRβ, platelet-derived growth factor receptor-β; VCAM-1, vascular cell adhesion molecule-1

Representative images show low PECAM-1 and VCAM-1 signal in no cSVD controls and increased signal in severe disease (Fig. 3a, b). PECAM-1 signal increased significantly with disease severity, displaying the highest value in the severe cSVD group, being 41% and 67% higher than in no cSVD controls in the grey and white matter, respectively (p = 0.023 and 0.0002, respectively; Fig. 3c). VCAM-1, which is typically expressed at low levels in capillaries, followed a similar trend, with its signal more than doubling (134% increase, p = 0.0052 in grey matter; 155% increase, p = 0.012 in white matter; Fig. 3g).

This increase may reflect endothelial activation associated with ageing-related systemic inflammation (“inflamm-ageing”) or disease-specific vascular alterations. However, these changes did not show a clear linear relationship with either disease group or age (Pearson’s R = 0.348 and 0.513 in grey matter, and 0.406 and 0.551 in white matter for PECAM-1 and VCAM-1, respectively; Fig. 3d, h).

### PDGFRβ signal is heterogeneous and markedly reduced with increasing cSVD severity

PDGFRβ signal significantly decreased with increasing cSVD severity (Fig. 3a), ith markedly reduced signal in the most severely affected cases compared with no cSVD control (94% reduction, p = 0.0038 in grey matter; 87% reduction, p = 0.0327 in white matter; Fig. 3e). PDGFRβ signal was 52% lower in white matter than in grey matter in no cSVD controls, further emphasising the vulnerability of white matter.

Despite this difference between the tissues, PDGFRβ signal showed a strong negative correlation with both disease severity and age in grey (Pearson’s R = -0.694) and white (Pearson’s R = -0.581) matter (Fig. 3f).

PDGFRβ signal showed a heterogeneous distribution across cases, with several mild and moderate cSVD cases displaying markedly reduced signal. Given the modest cohort size and cross-sectional design, this pattern should not be interpreted as evidence of a discrete biological transition state.

### Gaussian mixture clustering identifies three clusters based on marker signals

**Fig. 4:**
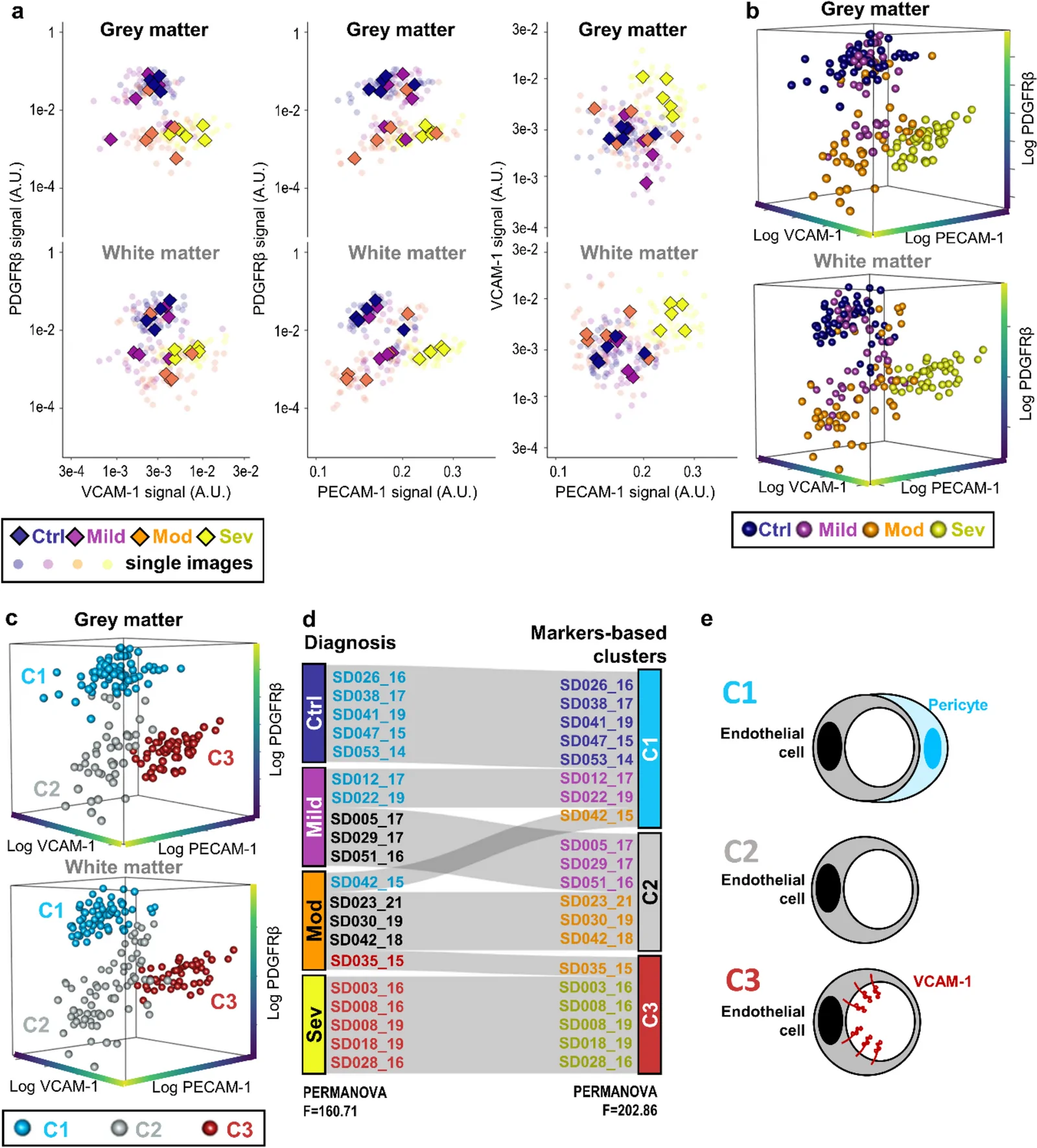
Marker-based clustering identifies distinct vascular marker phenotypes. **a** Pairwise scatterplots between log-transformed PECAM-1, PDGFRβ, and VCAM-1 signals. Diamond-shaped data points represent individual cases, whereas smaller dots represent analysed images. **b** 3D scatterplot of log-transformed PECAM-1, PDGFRβ and VCAM-1 signals; colours indicate diagnostic group and points represent analysed images. **c** 3D scatterplot of the same markers coloured according to marker-based clusters identified by Gaussian mixture modelling. **d** Distribution of cases from diagnostic categories (left) to marker-based clusters (right). PERMANOVA F-statistics were computed using the pool of analysed images to compare clustering strength by diagnostic group versus marker-defined clusters. **e** Schematic of the microvascular phenotypes associated with each cluster. C1 exhibited high PDGFRβ and low VCAM-1, C2 had low PDGFRβ and low VCAM-1, and C3 had low PDGFRβ and high VCAM-1. AU, arbitrary units; C1, cluster 1; C2, cluster 2; C3, cluster 3; cSVD, cerebral small vessel disease; Ctrl, no cSVD control; Mild, mild cSVD; Mod, moderate cSVD; Sev, severe cSVD; PECAM-1, platelet endothelial cell adhesion molecule-1; PDGFRβ, platelet-derived growth factor receptor-β; VCAM-1, vascular cell adhesion molecule-1

While pericyte loss is often associated with endothelial inflammation, there was no evidence in our data for a clear correlation between PDGFRβ loss and VCAM-1 or PECAM-1 increase. As visual inspection of the marker signal suggested the presence of underlying higher-order structure (Fig. 4a, b), we performed a Gaussian mixture clustering analysis, with a gap statistic indicating three clusters, to reveal data stratification. Three clusters were obtained by this method, designated cluster 1 (C1), cluster 2 (C2), and cluster 3 (C3) in subsequent analyses (Fig. 4c).

C1 was characterised by high PDGFRβ and low PECAM-1 and VCAM-1, C2 by low PDGFRβ with low PECAM-1 and VCAM-1, and C3 by low PDGFRβ combined with high PECAM-1 and VCAM-1 expression. These differences were statistically significant (Supplementary Fig. 1), and a higher PERMANOVA F-statistic was associated with the three clusters than with diagnostic alone (clusters: F = 202.86; diagnosis: F = 160.71), suggesting better separation of cases based on molecular marker profiles.

Intriguingly, the marker-based clustering largely recapitulated clinical diagnosis, with some notable deviations. C1 contained all no cSVD control cases, but also two cases from the mild group and one from the moderate group. C2 contained three cases from the mild group and three cases from the moderate group. C3 contained all severe cases, plus one from the moderate group (Fig. 4d).

This distribution broadly parallels increasing diagnostic severity but should not be interpreted as a temporal sequence. Rather, the clusters identify distinct microvascular marker phenotypes within this cross-sectional cohort **(**Fig. 4e).

### Circulating PDGF-BB and VCAM-1 profiles are associated with age

To complement the post-mortem observations with exploratory data from living patients, we analysed circulating PDGF-BB and VCAM-1 in a previously published lacunar stroke cohort. Overall, the V + /P- group was older than the V-/P + and V-/P- groups, whereas Fazekas score was not significantly different after adjustment for age and vascular risk factors. Mean age for the V + /P- quadrant (73.53 ± 8.83 y) was significantly higher than in the V-/P + (60.31 ± 10.6 y, p < 0.0001) and V-/P- (64.34 ± 11.14 y, p = 0.0032) quadrants (Supplementary Fig. 4b). However, Fazekas score, reflecting the extent of white matter damage, was not significant after correcting for patient age despite being highest in the V + /P- quadrant (3.79 ± 1.53, vs. 2.78 ± 1.59 in V-/P + and 2.89 ± 1.62 in V-/P-) (Supplementary Fig. 4c). These data therefore provide clinical context for the relationship between these circulating markers and age, but do not establish a temporal relationship between pericyte and endothelial changes.

## Discussion

We applied an automated vascular segmentation and analysis pipeline [9] to multispectral imaging of cortical brain samples in a cohort of 20 cases chosen to represent increasing cSVD severity, and quantified the vascular morphology and molecular markers associated with pericyte coverage and endothelial activation in relation to disease severity. In this cohort of 20 cases from Brodmann area 4, an understudied region in cSVD, we observed alterations in microglial morphology, reduced PDGFRβ signal and increased endothelial marker expression with increasing cSVD severity.

In contrast to previous reports showing age- and disease-related capillary density losses [7, 50], our study observed an increase in the capillary network density with worse SVD. However, we focused on superficial layers of one area of frontal cortex, while the heaviest burden of cSVD-related vascular remodelling is usually reported in deeper regions. White matter included here was proximate to the cortex, therefore perhaps better represents compensation than deep tissues. A preclinical study investigating capillary densities in superficial cortical layers of aged mice found a slight increase in density compared to younger animals [5], and a clinical study in AD cases revealed generally preserved vascular structure and even higher capillary density [15]. Capillary density increases have been discussed as evidence of endothelial remodelling in response to tissue hypoxia [31]. Our findings may therefore reflect a compensatory adaptation to underlying hypoxia or hypoperfusion in deeper regions.

Our additional Iba1 analysis also identified morphological differences in microglia in severe cSVD, including fewer and larger segmented Iba1-positive particles, consistent with reduced ramification. These findings support the involvement of microglia alongside vascular alterations in advanced cSVD, although the use of 4-µm sections precludes detailed reconstruction of microglial morphology or assessment of microglia-vascular interactions. More broadly, the contribution of other neurogliovascular cell populations, including astrocytes and oligodendroglial cells, and their relationships with pericyte and endothelial alterations will require further investigations.

Pericytes are thought to be key stabilisers of endothelial proliferation and vascular homeostasis in health [28], and our finding of reduced PDGFRβ signal with increasing cSVD severity is consistent with altered pericyte coverage. White and grey matter also exhibited significant differences in capillary morphology, as previously reported [17]. The lower density observed in the white matter further emphasises the vulnerability of this region to vascular-related damage. Although capillaries were slightly larger in white matter than in grey matter, we did not detect any significant change in the average diameter of capillaries across cSVD severity groups.

The overall trends in the vascular markers investigated in this study are broadly consistent with previous reports. CAMs mediate cell–cell interactions, including between endothelial cells and circulating immune cells, and an increase in their expression with disease burden [14, 26] is not surprising in cSVD due to its major inflammatory component. Pericytes are major regulators of the vascular basement membrane and endothelial stability, extending processes that form extensive networks around endothelial cells [25]. The two cell types share intricate relationships through gap junctions, peg-and-socket contacts, and paracrine communication, whose intensity is thought to be reflected by the relative area of contact between them [38]. PDGFRβ signal, used here as a measure of pericyte coverage, decreased with disease severity, supporting previous reports of pericyte loss in cSVD and other neurovascular dementias [13]. Pericyte loss also promotes white blood cell stalling by enhancing CAMs expression in endothelial cells [10].

Our analysis revealed heterogeneity that was not captured by a simple linear relationship between diagnostic severity and vascular marker expression. The distribution of PDGFRβ signal is an intriguing feature of this dataset, but its origin cannot be resolved in the present cohort. Inter-individual heterogeneity, regional variation, comorbid pathology, technical factors, and sampling may all contribute. Larger independent cohorts will be required to determine whether the observed distribution reflects a reproducible biological feature of cSVD.

As an exploratory approach to this heterogeneity, Gaussian mixture modelling identified three marker-defined clusters among the 20 cases distributed across four diagnostic severities of cSVD burden based on pathologist-determined microvessel sclerosis and related features. Cluster 1 (C1) was characterised by low CAM expression and high pericyte coverage, largely overlapping with young no cSVD controls. Cluster 2 (C2) displayed pericyte coverage loss while CAM expression remained low and grouped cases in the intermediate ‘mild’ and ‘moderate’ diagnostic categories. Cluster 3 (C3) presented both loss of pericyte coverage and the highest levels of endothelial CAM expression. These clusters should be regarded as descriptive molecular phenotypes rather than sequential disease states. Longitudinal and experimental studies will be required to determine whether these phenotypes are mechanistically related or occur in a defined temporal sequence.

There are several important limitations to this study. The relatively small post-mortem cohort (n = 20) limits statistical power, subgroup analyses, and the generalisability of the marker-defined clusters. The clustering should therefore be considered exploratory and requires replication in larger independent cohorts.

Furthermore, the absence of age-matched controls is a greater concern, as it partially confounds the distinction between cSVD-related changes and those associated with ageing. Importantly, identifying truly age-matched control individuals without some degree of WMH or microvascular pathology is increasingly difficult in older populations, which complicates the interpretation of vascular ageing in human cohorts. Two severe-cSVD cases also had cerebral amyloid angiopathy (CAA), which may independently affect pericytes, endothelial activation, and BBB integrity. Given the small number of CAA cases, we could not assess its contribution separately; therefore, CAA represents a potential confounder in the severe group and the corresponding findings should be interpreted cautiously.

The gender balance is also limited, with only 5 of 20 participants being female, which may affect the generalisability of our findings. Although cSVD increases with age, this distribution may still introduce bias in the interpretation of vascular alterations, and sex differences in clinical presentations have been reported [24]. Future studies should examine other brain regions in larger cohorts and ideally include age- and sex-matched non-cSVD controls.

Additionally, despite the diagnosis of mild-to-moderate cSVD, three cases showed relatively high pericyte coverage, possibly reflecting regional resilience of the vasculature within the analysed cortical region. The diagnostic groups used in this study reflect neuropathological cSVD severity rather than validated temporal stages of disease. cSVD lacks a universally accepted neuropathological staging system, and the cross-sectional ordering of cases by severity cannot establish individual disease trajectories. Accordingly, differences between groups should be interpreted as associations with cSVD burden rather than evidence of sequential pathological events.

Another important limitation of the present study is its restricted anatomical scope. The available tissue blocks were obtained from Brodmann area 4 and predominantly sampled the superficial white matter immediately beneath the cortex, whereas cSVD pathology is regionally heterogeneous and frequently most pronounced in the deep white matter. Our findings should therefore not be assumed to generalise to true deep white matter or other brain regions affected by cSVD. An internal comparison of superficial and relatively deeper white matter within the available blocks showed similar vascular measurements (data not shown); however, this does not substitute for analysis of true deep white matter. Future studies incorporating larger tissue blocks and multiple brain regions will be required to determine the regional generalisability of the vascular phenotypes identified here.

Because the present study is based on post-mortem marker expression, it does not directly assess BBB permeability, tight junction integrity, pericyte viability, or vascular physiology. Accordingly, the observed PDGFRβ and endothelial marker changes should not be interpreted as direct functional measurements. Future studies incorporating CLDN5, endogenous leakage markers such as IgG and fibrinogen, and functional experimental systems will be required to link these molecular phenotypes to BBB dysfunction. In addition, the contribution of other NGVU cell types, including astrocytes, microglia, and oligodendroglial populations, warrants investigation, as these cells are likely to interact with pericytes and endothelial cells to modulate microvascular dysfunction in cSVD.

Our findings identify heterogeneous pericyte and endothelial marker profiles in human cSVD and show that these features do not vary in a simple parallel manner across neuropathological severity. These cross-sectional observations provide a framework for future longitudinal and experimental studies to determine the temporal and mechanistic relationships between pericyte alterations, endothelial activation, and BBB dysfunction.

## Supplementary Information

Below is the link to the electronic supplementary material.

Supplementary Material 1.

## Data Availability

Data will be made available upon reasonnable request.

## References

[1] Anderle S, Dixon M, Quintela-Lopez T, Sideris-Lampretsas G, Attwell D (2025) The vascular contribution to cognitive decline in ageing and dementia. Nat Rev Neurosci. 10.1038/s41583-025-00950-1 40760183

[2] Armulik A, Genové G, Mäe M, Nisancioglu MH, Wallgard E, Niaudet C et al (2010) Pericytes regulate the blood–brain barrier. Nature 468:557–561. 10.1038/nature09522 20944627

[3] Bates D, Maechler M, Bolker [aut B, Walker S, Christensen RHB, Singmann H, et al. simulate.formula) PNK (shared copyright on, Tanaka E, Jagan M, Boylan RD, Ly A (2025) lme4: Linear Mixed-Effects Models using “Eigen” and S4

[4] Bell RD, Winkler EA, Sagare AP, Singh I, LaRue B, Deane R et al (2010) Pericytes control key neurovascular functions and neuronal phenotype in the adult brain and during brain aging. Neuron 68:409–427. 10.1016/j.neuron.2010.09.043 PMC3056408 21040844

[5] Bennett HC, Zhang Q, Wu Y, Manjila SB, Chon U, Shin D et al (2024) Aging drives cerebrovascular network remodeling and functional changes in the mouse brain. Nat Commun 15:6398. 10.1038/s41467-024-50559-8 PMC11289283 39080289

[6] Blair GW, Doubal FN, Thrippleton MJ, Marshall I, Wardlaw JM (2016) Magnetic resonance imaging for assessment of cerebrovascular reactivity in cerebral small vessel disease: a systematic review. J Cereb Blood Flow Metab 36:833–841. 10.1177/0271678X16631756 PMC4853842 26884471

[7] Brown WR, Moody DM, Thore CR, Challa VR, Anstrom JA (2007) Vascular dementia in leukoaraiosis may be a consequence of capillary loss not only in the lesions, but in normal-appearing white matter and cortex as well. J Neurol Sci 257:62–66. 10.1016/j.jns.2007.01.015 PMC1989116 17320909

[8] Chagnot A, Montagne A (2025) The blood–brain barrier. Curr Biol 35:R1010–R1015. 10.1016/j.cub.2025.06.061 41118727

[9] Chagnot A, Montagne A (2025) Morph-O-Matic: A MATLAB-based tool for batch cell segmentation and measurement on 2D microscopy images

[10] Choe Y-G, Yoon J-H, Joo J, Kim B, Hong SP, Koh GY et al (2022) Pericyte loss leads to capillary stalling through increased leukocyte-endothelial cell interaction in the brain. Front Cell Neurosci 16:848764. 10.3389/fncel.2022.848764 PMC8962364 35360491

[11] Daneman R, Zhou L, Kebede AA, Barres BA (2010) Pericytes are required for blood–brain barrier integrity during embryogenesis. Nature 468:562–566. 10.1038/nature09513 PMC3241506 20944625

[12] Deary IJ, Gow AJ, Taylor MD, Corley J, Brett C, Wilson V et al (2007) The Lothian birth cohort 1936: a study to examine influences on cognitive ageing from age 11 to age 70 and beyond. BMC Geriatr 7:28. 10.1186/1471-2318-7-28 PMC2222601 18053258

[13] Ding R, Hase Y, Ameen-Ali KE et al (2020) Loss of capillary pericytes and the blood–brain barrier in white matter in poststroke and vascular dementias and Alzheimer’s disease. Brain Pathol 30:1087–1101. 10.1111/bpa.12888 PMC8018063 32705757

[14] Fassbender K, Bertsch T, Mielke O, Mühlhauser F, Hennerici M (1999) Adhesion Molecules in Cerebrovascular Diseases. Stroke 30:1647–1650. 10.1161/01.STR.30.8.1647 10436116

[15] Fernandez-Klett F, Brandt L, Fernández-Zapata C, Abuelnor B, Middeldorp J, Sluijs JA et al (2020) Denser brain capillary network with preserved pericytes in Alzheimer’s disease. Brain Pathol 30:1071–1086. 10.1111/bpa.12897 PMC8018033 32876357

[16] Hall CN, Reynell C, Gesslein B, Hamilton NB, Mishra A, Sutherland BA et al (2014) Capillary pericytes regulate cerebral blood flow in health and disease. Nature 508:55–60. 10.1038/nature13165 PMC3976267 24670647

[17] Hase Y, Ding R, Harrison G, Hawthorne E, King A, Gettings S et al (2019) White matter capillaries in vascular and neurodegenerative dementias. Acta Neuropathol Commun 7:16. 10.1186/s40478-019-0666-x PMC6366070 30732655

[18] Hase Y, Jobson D, Cheong J, Gotama K, Maffei L, Hase M et al (2024) Hippocampal capillary pericytes in post-stroke and vascular dementias and Alzheimer’s disease and experimental chronic cerebral hypoperfusion. Acta Neuropathol Commun 12:29. 10.1186/s40478-024-01737-8 PMC10870440 38360798

[19] Hristovska I, Pichet Binette A, Kumar A, Wennström M, Gaiteri C, Karlsson L et al (2026) Identification of distinct and shared biomarker panels in different manifestations of cerebral small-vessel disease through proteomic profiling. Nat Aging. 10.1038/s43587-026-01081-7 PMC13004690 41735646

[20] Humphreys CA, Jansen MA, Muñoz Maniega S, González-Castro V, Pernet C et al (2019) A protocol for precise comparisons of small vessel disease lesions between ex vivo magnetic resonance imaging and histopathology. Int J Stroke 14:310–320. 10.1177/1747493018799962 PMC6604680 30196792

[21] Iadecola C (2013) The pathobiology of vascular dementia. Neuron 80:844–866. 10.1016/j.neuron.2013.10.008 PMC3842016 24267647

[22] Jaime Garcia D, Chagnot A, Wardlaw JM, Montagne A (2023) A scoping review on biomarkers of endothelial dysfunction in small vessel disease: molecular insights from human studies. Int J Mol Sci 24:13114. 10.3390/ijms241713114 PMC10488088 37685924

[23] Jaime Garcia D, Clancy U, Arteaga C, Valdés-Hernandez MC, Chappell FM et al (2025) Blood biomarkers of vascular dysfunction in small vessel disease progression: Insights from a longitudinal neuroimaging study. Alzheimer’s Dementia 21:e70152. 10.1002/alz.70152 PMC12022501 40275856

[24] Jiménez-Sánchez L, Hamilton OKL, Clancy U, Backhouse EV, Stewart CR, Stringer MS et al (2021) Sex differences in cerebral small vessel disease: a systematic review and meta-analysis. Front Neurol 12:756887. 10.3389/fneur.2021.756887 PMC8581736 34777227

[25] Kovacs-Oller T, Ivanova E, Bianchimano P, Sagdullaev BT (2020) The pericyte connectome: spatial precision of neurovascular coupling is driven by selective connectivity maps of pericytes and endothelial cells and is disrupted in diabetes. Cell Discov 6:1–18. 10.1038/s41421-020-0180-0 PMC7296038 32566247

[26] Kremer R, Williams A, Wardlaw J (2025) Endothelial cells as key players in cerebral small vessel disease. Nat Rev Neurosci 26:179–188. 10.1038/s41583-024-00892-0 39743557

[27] Lenth RV, Piaskowski J, Banfai B, Bolker B, Buerkner P, Giné-Vázquez I, et al (2025) emmeans: Estimated Marginal Means, aka Least-Squares Means

[28] Li G, Gao J, Ding P, Gao Y (2025) The role of endothelial cell–pericyte interactions in vascularization and diseases. J Adv Res 67:269–288. 10.1016/j.jare.2024.01.016 PMC11725166 38246244

[29] van der Loos CM (2010) Chromogens in multiple immunohistochemical staining used for visual assessment and spectral imaging: the colorful future. J Histotechnol 33:31–40. 10.1179/his.2010.33.1.31

[30] Mäe MA, He L, Nordling S, Vazquez-Liebanas E, Nahar K, Jung B et al (2021) Single-cell analysis of blood-brain barrier response to pericyte loss. Circ Res 128:e46–e62. 10.1161/CIRCRESAHA.120.317473 PMC10858745 33375813

[31] Manning D, Rivera EJ, Santana LF (2024) The life cycle of a capillary: mechanisms of angiogenesis and rarefaction in microvascular physiology and pathologies. Vasc Pharmacol 156:107393. 10.1016/j.vph.2024.107393 PMC12051481 38857638

[32] Markus HS, de Leeuw FE (2023) Cerebral small vessel disease: recent advances and future directions. Int J Stroke 18:4–14. 10.1177/17474930221144911 PMC9806465 36575578

[33] Miners JS, Kehoe PG, Love S, Zetterberg H, Blennow K (2019) CSF evidence of pericyte damage in Alzheimer’s disease is associated with markers of blood-brain barrier dysfunction and disease pathology. Alzheimers Res Ther 11:81. 10.1186/s13195-019-0534-8 PMC6745071 31521199

[34] Miners JS, Schulz I, Love S (2018) Differing associations between Aβ accumulation, hypoperfusion, blood–brain barrier dysfunction and loss of PDGFRB pericyte marker in the precuneus and parietal white matter in Alzheimer’s disease. J Cereb Blood Flow Metab 38:103–115. 10.1177/0271678X17690761 PMC5757436 28151041

[35] Montagne A, Barnes SR, Sweeney MD, Halliday MR, Sagare AP, Zhao Z et al (2015) Blood-brain barrier breakdown in the aging human hippocampus. Neuron 85:296–302. 10.1016/j.neuron.2014.12.032 PMC4350773 25611508

[36] Nation DA, Sweeney MD, Montagne A, Sagare AP, D’Orazio LM, Pachicano M et al (2019) Blood-brain barrier breakdown is an early biomarker of human cognitive dysfunction. Nat Med 25:270–276. 10.1038/s41591-018-0297-y PMC6367058 30643288

[37] Nikolakopoulou AM, Montagne A, Kisler K, Dai Z, Wang Y, Huuskonen MT et al (2019) Pericyte loss leads to circulatory failure and pleiotrophin depletion causing neuron loss. Nat Neurosci 22:1089–1098. 10.1038/s41593-019-0434-z PMC6668719 31235908

[38] Ornelas S, Berthiaume A-A, Bonney SK, Coelho-Santos V, Underly RG, Kremer A et al (2021) Three-dimensional ultrastructure of the brain pericyte-endothelial interface. J Cereb Blood Flow Metab 41:2185–2200. 10.1177/0271678X211012836 PMC8393306 33970018

[39] Otsu N (1979) A threshold selection method from gray-level histograms. IEEE Trans Syst Man Cybern 9:62–66. 10.1109/TSMC.1979.4310076

[40] Pantoni L (2010) Cerebral small vessel disease: from pathogenesis and clinical characteristics to therapeutic challenges. Lancet Neurol 9:689–701. 10.1016/S1474-4422(10)70104-6 20610345

[41] Procter TV, Williams A, Montagne A (2021) Interplay between brain pericytes and endothelial cells in dementia. Am J Pathol 191:1917–1931. 10.1016/j.ajpath.2021.07.003 PMC12179519 34329605

[42] Samarasekera N, Lerpiniere C, Fonville AF, Farrall AJ, Wardlaw JM, White PM et al (2015) Consent for Brain Tissue Donation after Intracerebral Haemorrhage: A Community-Based Study. PLoS ONE 10:e0135043. 10.1371/journal.pone.0135043 PMC4547774 26302447

[43] Sengillo JD, Winkler EA, Walker CT, Sullivan JS, Johnson M, Zlokovic BV (2013) Deficiency in mural vascular cells coincides with blood-brain barrier disruption in Alzheimer’s disease. Brain Pathol 23:303–310. 10.1111/bpa.12004 PMC3628957 23126372

[44] Sewell M, Fialova N, Montagne A (2025) Unraveling the transcriptomic landscape of brain vascular cells in dementia: a systematic review. Alzheimers Dement 21:e14512. 10.1002/alz.14512 PMC11851133 39807599

[45] Skrobot OA, Attems J, Esiri M, Hortobágyi T, Ironside JW, Kalaria RN et al (2016) Vascular cognitive impairment neuropathology guidelines (VCING): the contribution of cerebrovascular pathology to cognitive impairment. Brain 139:2957–2969. 10.1093/brain/aww214 27591113

[46] Smith C, Millar T (2018) Brain donation procedures in the sudden death brain bank in Edinburgh. Handb Clin Neurol 150:17–27. 10.1016/B978-0-444-63639-3.00002-5 29496140

[47] Sweeney MD, Zhao Z, Montagne A, Nelson AR, Zlokovic BV (2019) Blood-brain barrier: from physiology to disease and back. Physiol Rev 99:21–78. 10.1152/physrev.00050.2017 PMC6335099 30280653

[48] Thrippleton MJ, Backes WH, Sourbron S, Ingrisch M, van Osch MJP, Dichgans M et al (2019) Quantifying blood-brain barrier leakage in small vessel disease: review and consensus recommendations. Alzheimers Dement 15:840–858. 10.1016/j.jalz.2019.01.013 PMC6565805 31031101

[49] Török O, Schreiner B, Schaffenrath J, Tsai H-C, Maheshwari U, Stifter SA et al (2021) Pericytes regulate vascular immune homeostasis in the CNS. Proc Natl Acad Sci 118:e2016587118. 10.1073/pnas.2016587118 PMC7958247 33653955

[50] Voorter PH, van Dinther M, Drenthen GS, Elschot EP, Staals J, van Oostenbrugge RJ et al (2025) Vessel architecture imaging reveals microvascular rarefaction and capillary-to-arteriole shift in cerebral small vessel disease. J Cereb Blood Flow Metab 45:2092–2103. 10.1177/0271678X251358968 PMC12316679 40745215

[51] Wardlaw JM, Smith C, Dichgans M (2019) Small vessel disease: mechanisms and clinical implications. Lancet Neurol 18:684–696. 10.1016/S1474-4422(19)30079-1 31097385

[52] Wardlaw JM, Woodhouse LJ, Mhlanga II, Oatey K, Heye AK, Bamford J et al (2023) Isosorbide Mononitrate and Cilostazol Treatment in Patients With Symptomatic Cerebral Small Vessel Disease: The Lacunar Intervention Trial-2 (LACI-2) Randomized Clinical Trial. JAMA Neurol 80:682–692. 10.1001/jamaneurol.2023.1526 PMC10209826 37222252

[53] Yousef H, Czupalla CJ, Lee D, Chen MB, Burke AN, Zera KA et al (2019) Aged blood impairs hippocampal neural precursor activity and activates microglia via brain endothelial cell VCAM1. Nat Med 25:988–1000. 10.1038/s41591-019-0440-4 PMC6642642 31086348

[54] Zanier ER, Fumagalli S, Perego C, Pischiutta F, De Simoni M-G (2015) Shape descriptors of the “never resting” microglia in three different acute brain injury models in mice. Intensive Care Med Exp 3:7. 10.1186/s40635-015-0039-0 PMC4513020 26215806

