# Supplementary material for: Pericyte loss and endothelial activation define distinct vascular states in cerebral small vessel disease
Source: Acta Neuropathol. 2026 Oct 13;152(1):53. doi: 10.1007/s00401-026-03102-6 (PMC13661683; doi:10.1007/s00401-026-03102-6)
Supplement: Supplementary file 1 [file 401_2026_3102_MOESM1_ESM.docx]

# Supplementary Tables and Figures

**Supplementary Table 1. Biomarkers cohort characteristics.** Abbreviations: SD, standard deviation; IQR, interquartile range; PDGF-BB, platelet derived growth factor-BB; VCAM-1, vascular cell adhesion molecule-1.

**Supplementary Figure 1. Multispectral imaging and spectral unmixing workflow.** Brightfield images were decomposed into marker-specific channels using acquisitions across 20 wavelengths. Reference spectra were obtained from single-stained tissues from the same cohort, allowing separation of individual chromogenic signals, including signals that are difficult to distinguish visually. This approach also avoids limitations associated with autofluorescence in aged post-mortem human tissue.

**Supplementary Figure 2. Microglial morphology across cSVD severity groups.** (**a**) Representative Iba1-positive microglia from no cSVD control, mild, moderate, and severe cSVD cases. (**b**) Illustrative Iba1 segmentation presenting the image-analysis approach used to quantify microglial features in 4-µm tissue sections. (**c**) Iba1-positive area in grey and white matter across cSVD severity groups. (**d**) Number of segmented Iba1-positive particles in grey and white matter across cSVD severity groups. (**e**) Median area of segmented Iba1-positive particles in grey and white matter across cSVD severity groups. Post-hoc comparisons were performed using estimated marginal means (emmeans) to test differences between cSVD severity groups (p < 0.05). Abbreviations: cSVD, cerebral small vessel disease; Ctrl, no cSVD control; Mild, mild cSVD; Mod, moderate cSVD; Sev, severe cSVD; Iba1, ionized calcium-binding adaptor molecule 1.

**Supplementary Figure 3. Vascular density, vascular diameter, PECAM-1, PDGFRβ, and VCAM-1 signals across marker-based clusters.** Linear mixed-effects modelling was used to account for case-level variability, with tissue compartment (grey/white matter) and images included as hierarchical factors. (**a**) Vascular density, (**b**) vascular diameter, (**c**) PECAM-1 signal, (**d**) PDGFRβ signal, and (**e**) VCAM-1 signal. Post-hoc comparisons were performed using estimated marginal means (emmeans) and detected significant changes between clusters (*, p-value < 0.05; **, p-value < 0.01; ***, p-value < 0.001). Abbreviations: AU, arbitrary units; C1, cluster 1; C2, cluster 2; C3, cluster 3; PECAM-1, platelet endothelial cell adhesion molecule-1; PDGFRβ, platelet-derived growth factor receptor-β; VCAM-1, vascular cell adhesion molecule-1.

**Supplementary Figure 4. Blood biomarkers analysis reveals associations of circulating PDGF-BB and VCAM-1 with age.** (**a**) Individual patient readouts of blood PDGF-BB and VCAM-1. Half-maximal PDGF-BB and VCAM-1 values were used to define four exploratory quadrants: low VCAM-1 / high PDGF-BB (V-/P+, n=41), low VCAM-1 / low PDGF-BB (V-/P-, n=99), high VCAM-1 / low PDGF-BB (V+/P-, n=34) and high VCAM-1 / high PDGF-BB (V+/P+, n=5). The V+/P+ group was excluded from subsequent group comparisons because of its small size. (**b**) Age was significantly higher in the V+/P- quadrant. (**c**) Fazekas score was not significantly different between the three main quadrants after adjustment. Post-hoc comparisons were performed using estimated marginal means (emmeans) from regression models. Age comparisons were adjusted for hypertension, diabetes, and hypercholesterolaemia, while Fazekas score comparisons were additionally adjusted for age (n.s., not significant; **, p-value < 0.01; ***, p-value < 0.001). Abbreviations: PDGF-BB: platelet-derived growth factor BB, VCAM-1, vascular cell adhesion molecule-1.
